# Editorial: Central Asian medicinal plants and fungi as sources of biologically active extracts and metabolites

**DOI:** 10.3389/fphar.2024.1520143

**Published:** 2024-11-20

**Authors:** Ana Hortência Fonsêca Castro, Anna Malm, Dmitriy Berillo

**Affiliations:** ^1^ Universidade Federal de São João del-Rei, São João del Rei, Brazil; ^2^ Medical University of Lublin, Lublin, Poland; ^3^ Satbayev University, Almaty, Kazakhstan

**Keywords:** saponins, Central Asia, *Solanum*, herbal medicine, medicinal plant

Central Asia, including such countries as Kazakhstan, Kyrgyzstan, Tajikistan, Turkmenistan and Uzbekistan, has a long and rich history of traditional medicine. However, in ethnopharmacological terms this geographical region is one of the least known globally.

This Research Topic aimed at understanding the chemical diversity of medicinal plants and fungi in this important region. Another objective was to reveal novel and optimised methods of extraction of biologically active compounds, and also separation of compounds by classes, in order to illustrate possible applications of extracts via preparation of formulations, evaluation of activity *in vivo* and *in vitro*, and estimation of acute and chronic toxicity.

The flora of Central Asia contains thousands of plant and fungal species, both endemic and widely distributed; many of them have been used as remedies in traditional medicine. *Stachys sylvatica* L. (Lamiaceae) occurs in both hemispheres. Studies performed by Mukhamedsadykova et al. on hydroethanolic extract of this plant species growing in Kazakhstan indicated that its chemical profile differred partly from the extracts obtained from specimen occurring in Europe. Besides, an interesting anthelmintic activity of the studied extract was revealed for first time during the *in vitro* bioactivity research, being a prerequisite for further antiparasitic studies.

In this context, Gafforov et al. reviewed the biodiversity and ethnobotanical significance of eight native and non-native *Solanum* species in Uzbekistan revealing the cultural wealth and ethnopharmacological uses of *S. dulcamara* L., *S. lycopersicum* L., *S. melongena* L., *S. nigrum* L., *S. rostratum* Dunal., *S. sisymbriifolium* Lam., *S. tuberosum* L., and *S. villosum* Mill. The authors presented data on the diversity, morphological characteristics, global distribution, habitat, population status, phenology, reproduction, pharmacology and phytochemistry of these *Solanum* species. This highlights the significance of continued phytochemical research to maximize the medicinal potential of *Solanum* species. The findings provides important insights for future investigations and the creation of new pharmaceutical innovations.

Extracts of an herbal medicine from Asian medicinal plants also exhibit antitumor activity. Liu et al. showed the molecular mechanism of the Chebulae Fructus (CFE), a common herbal medicine in Asia medicine related to inhibitory effects on Hepatocellular carcinoma (HCC). Authors evaluated the anti-HCC effect of the aqueous extract of CFE on human HCC and its underlying mechanism, demonstrating that CFE effectively suppressed the proliferation and activity of HepG2 and PLC/PRF/5 HCC cells. CFE also induced apoptosis, and suppressed the migration and invasion abilities of these cells and exhibited inhibitory effects on tumor growth.

The effects of saponins from *Polygala tenuifolia* Willd (Polygalaceae) on dementia were widely discussed and reviewed by Li et al., providing experimental evidence and new insights for the research and application of saponins in this field. In this review, the authors presented the saponin components of *P. tenuifolia*, including tenuigenin, tenuifolin, polygalasaponins XXXII, and onjisaponin B and the potential mechanisms by which the active components of *P. tenuifolia* prevent and treat diseases based on relevant clinical triels. Combining saponin compounds from *Polygala tenuifolia* with current treatment protocols could provide an innovative strategy for slowing the progression of neurodegenerative diseases.

Finally, Li et al. explored the inhibitory effect of a new type of polysaccharide isolated and extracted from pomegranate flowers (PFPS) on mastitis in *in vitro* and *in vivo* models. The results indicate that PFPS can effectively prevent mastitis by regulating the intestinal flora of mice, reducing the relative abundance of pathogenic bacteria, and increasing the probiotics *Blautia*, *Parabacteroides*, *Allobaculum*, and *Clostridiaceae_Clostridium* by improving the blood-milk barrier. This investigation provided a scientific basis for PFPS as a potential candidate drug for the treatment of mastitis.

In conclusion, biological and pharmacologial activities of several plant remedies used in traditional medicine in Central Asia have been reviewed or confirmed in the above mentioned articles, while providing novel data being rationale for further studies.

